# Phylogenetic analysis of emergent *Streptococcus pneumoniae* serotype 22F causing invasive pneumococcal disease using whole genome sequencing

**DOI:** 10.1371/journal.pone.0178040

**Published:** 2017-05-22

**Authors:** Walter H. B. Demczuk, Irene Martin, Linda Hoang, Paul Van Caeseele, Brigitte Lefebvre, Greg Horsman, David Haldane, Jonathan Gubbay, Sam Ratnam, Gregory German, Jennifer Daley Bernier, Lori Strudwick, Allison McGeer, George G. Zhanel, Gary Van Domselaar, Morag Graham, Michael R. Mulvey

**Affiliations:** 1 National Microbiology Laboratory, Public Health Agency of Canada, Winnipeg, Manitoba, Canada; 2 British Columbia Centre for Disease Control, Vancouver, British, Canada; 3 Cadham Provincial Laboratory, Winnipeg, Manitoba, Canada; 4 Laboratoire de santé publique du Québec, Ste-Anne-de-Bellevue, Québec, Canada; 5 Saskatchewan Disease Control Laboratory, Regina, Saskatchewan, Canada; 6 Queen Elizabeth II Health Science Centre, Halifax, Nova Scotia, Canada; 7 Public Health Ontario, Toronto, Ontario, Canada; 8 Newfoundland Public Health Laboratory, St. John’s, Newfoundland and Labrador, Canada; 9 Queen Elizabeth Hospital, Charlottetown, Prince Edward Island, Canada; 10 Stanton Territorial Hospital Laboratory, Yellowknife, Northwest Territories, Canada; 11 Whitehorse General Hospital, Whitehorse, Yukon, Canada; 12 Toronto Invasive Bacterial Diseases Network, Mount Sinai Hospital, Toronto, Ontario, Canada; 13 Department of Medical Microbiology and Infectious Diseases, University of Manitoba, Winnipeg, Manitoba, Canada; 14 Science Technology Cores and Services Division, National Microbiology Laboratory, Public Health Agency of Canada, Winnipeg, Manitoba, Canada; Instituto Butantan, BRAZIL

## Abstract

Since implementation of the 13-valent polyvalent conjugate vaccine (PCV13) in Canada during 2010, the proportion of PCV13 serotypes causing invasive pneumococcal disease (IPD) has declined from 55% (*n* = 1492) in 2010 to 31% (*n* = 764) in 2014. A concurrent increase of non-PCV13 serotypes has occurred and 22F has become the most prevalent serotype in Canada increasing from 7% (*n* = 183) to 11% (*n* = 283). Core single nucleotide variant phylogenetic analysis was performed on 137 *Streptococcus pneumoniae* serotype 22F isolates collected across Canada from 2005–2015. Six phylogenetic lineages (*n* = 117) were identified among a serotype 22F/ST433 clonal complex (CC), including a recently expanding erythromycin-resistant clone. Erythromycin-resistance was observed in 25 isolates possessing *ermB*, *mef* or a 23S rRNA A2061G point mutation; 2 penicillin-resistant isolates had recombinant *pbp1a*, *pbp2a* and/or *pbp2x*; 3 tetracycline-resistant isolates contained *tetM*; and 1 isolate was multidrug-resistant. Virulence factor analysis indicated a high level of homogeneity among the 22F/ST433 clonal complex strains. A group of 6 phylogenetic outlier strains had differing MLST, antimicrobial resistance and molecular profiles suggestive of capsule switching events. While capsule switch events among *S*. *pneumoniae* serotype 22F has been observed, increasing prevalence of *S*. *pneumoniae* serotype 22F can be attributed to an evolving homogenous clone expanding nationally through local transmission events.

## Introduction

*Streptococcus pneumoniae*, a Gram-positive commensal organism of the human nasopharynx, is a major cause of community-acquired pneumonia as well as severe invasive pneumococcal disease (IPD) such as bacteremia and meningitis. Pneumococcal disease is associated with a heavy burden of mortality and morbidity, globally causing an estimated 1.6 million deaths a year, with more than half represented by children <5 years of age [1]. IPD represented the largest proportion of diseases transmitted by respiratory routes (45.8%, *n* = 3,178) in Canada during 2014 [2].

The pneumococcal capsule polysaccharide (CPS) is a primary virulence factor and antigen with 97 currently recognized serotypes [3], of which only a small subset cause the majority of disease. Current pneumococcal conjugate vaccines targeting the prevalent serotypes have been effective in directly lowering vaccine serotypes of IPD in children as well as conferring indirect herd immunity to other age groups [4,5]. Since the implementation of the 13-valent polyvalent conjugate vaccine (PCV13) in Canada starting in 2010, the proportion of IPD caused by constituent serotypes (4, 6B, 9V, 14, 18C, 19F, 23F, 1, 3, 5, 7A, 7F and 19A) has declined from 55% (*n* = 1,492) in 2010 to 31% (*n* = 764) in 2014 (p<0.05, Odds Ratio (OR) = 2.7, Confidence Interval at 95% (CI) = 2.45–3.08) [6]. Despite similar reductions worldwide, a concurrent increase of disease caused by non-vaccine serotypes (NVTs) has been reported [7–12] with serotype 22F being a major contributor [13–21]. In Canada from 2010–2014, NVTs increased from 45% (*n* = 1,216) to 69% (*n* = 1,709) (p<0.05, OR = 2.7, CI = 2.45–3.08) with serotype 22F increasing from 7% (*n* = 183) to 11% (*n* = 283) (p<0.05, OR = 0.56, CI = 0.46–0.68) [6]. This increase has been broadly based among all age groups and is the most prevalent serotype in all regions of Canada (S1 Fig). Although antimicrobial resistance among serotype 22F strains has generally been low in Canada [6], elevated disease severity associated with this serotype is cause for concern [22,23]. Although serotype 22F is a component of the 23-valent pneumococcal polysaccharide vaccine (PPV23) it is not licenced for routine use in young children and has little efficacy on carriage, an important reservoir for the transmission and spread of invasive disease [13,16,17,19,23]. PPV23 has also had little effect on IPD prevalence rates of constituent serotypes in adults, possibly due to low coverage or efficacy [13,19,24,25].

The emergence and spread of NVTs can occur either by compensatory clonal expansion of a minor circulating strain after eradication of specific predominant vaccine serotypes, or by capsule switching events through horizontal recombination of the CPS coding region among colonizing strains [7,10,11,26,27]. Traditional molecular characterization techniques that consider evolutionary dynamics, such as multi-locus sequence typing (MLST), have been useful in identifying capsule switching events [7,28]. Whole-genome sequencing (WGS) techniques can further enhance this resolution to infer genetic relatedness for better understanding the emergence, dissemination and dynamics of pneumococci [28,29].

In this study we describe the distribution of increasingly prevalent *S*. *pneumoniae* serotype 22F strains in Canada by characterization of MLST sequence types (ST), antimicrobial resistance (AMR) determinants, and virulence factors using WGS analyses.

## Materials and methods

### Isolates and antimicrobial susceptibility testing

A total of 112 invasive Canadian *S*. *pneumoniae* serotype 22F isolates collected in Canada from 2005–2015 were selected for analysis, representing approximately 7% of nationally reported invasive serotype 22F (S1 Table). Approximately 5 isolates from each region (Western, Central and Eastern Canada) for each year from 2010–2014 were randomly selected from a list of sample identification numbers to ensure an even geographical and temporal distribution of strains. Fewer invasive serotype 22F isolates were available prior to the start of national surveillance from 2005–2009 (*n* = 7); and for the period from January 1 to March 31, 2015 (*n* = 10). Canadian invasive strains were compared to respiratory 22F isolates (*n* = 25), other miscellaneous serotypes (*n* = 58), invasive serotype 22F genomes from the USA (*n* = 21, NCBI BioProject PRJEB2632) [29] and Pneumococcal Molecular Epidemiology Network (PMEN) clone genomes (*n* = 26, University of Manchester, NCBI BioProject PRJEB10893).

Antimicrobial susceptibilities were determined using Sensititre^TM^ STP6F micro-broth dilution panels (Thermo Fisher^TM^, USA) and resistant (R), intermediate (I) or susceptible (S) interpretations of minimum inhibitory concentration (MIC) for erythromycin (ERY), clindamycin (CLI), penicillin (PEN), cefepime (CFM), cefotaxime (CEF), ceftriaxone (CRO), meropenem (MER), trimethoprim/sulfamethoxazole (SXT) and tetracycline (TET) were determined using Clinical Laboratory Standards Institute guidelines [30]. Meningitis resistance breakpoints were used for PEN, CFM, CEF and CRO. Multi-drug resistance (MDR) was defined as resistance to 3 or more classes of antimicrobials (ß-lactams, macrolides, tetracycline and trimethoprim/sulfamethoxazole).

### Whole-genome sequencing and assembly

DNA samples were extracted from cultures following standard protocol with Epicentre Masterpure Complete DNA and RNA Extraction Kit (Mandel Scientific, Guelph, ON). Multiplexed libraries were created with TruSeq sample preparation kits (Illumina, San Diego, CA) and paired-end, 300 bp indexed reads were generated on the Illumina MiSeq platform (Illumina, San Diego, CA). WGS read data were submitted to the NCBI Short Read Archive under BioProject PRJNA347910. The quality of the reads was assessed using FastQC version 0.11.4 (http://www.bioinformatics.babraham.ac.uk/projects/fastqc/), merged using FLASH version 1.2.9 with minimum overlap = 20 and maximum overlap = 300 [31], assembled with SPAdes version 3.6.2 [32] and annotated with Prokka version 1.11 [33].

### Core single nucleotide variation (SNV) phylogenetic analysis

FASTQ forward and reverse read files were analyzed using a custom Galaxy SNVphyl paired end fastq workflow (https://github.com/phac-nml/snvphyl-galaxy) with minimum coverage = 15, minimum mean mapping quality = 30, and alternative allele ratio = 0.75. The high-quality reads were then mapped to the publically available reference genome, *S*. *pneumoniae* R6 (NCBI Accession NC_003098.1) with SMALT version 0.7.5 (http://www.sanger.ac.uk/resources/software/smalt/) with smalt index K-mer size set to 13 and Step size to 6; and smalt map maximum insert size = 1000, minimum insert size = 20, seed = 1, and identity threshold = 0.5. Single nucleotide variants were called using FreeBayes (Erik Garrison, Garbor Marth (2012) arXiv:1207.3907[q-bio.GN]) using the following parameters: “—theta 0.001—pvar 0—ploidy 1—left-align-indels—min-mapping-quality 30—min-base-quality 30—min-alternate-fraction 0.75—min-coverage 15” with additional variant confirmation using SAMtools mpileup [34] and positions where variant calls were not in agreement between both variant callers were excluded. Variant calls within potential problematic regions including repetitive regions identified with Mummer version 3.23) with minimum length of repeat region set to 150 and minimum PID of repeat region to 90 and highly recombinant regions containing >10 SNVs per 100 base pairs were removed from the analysis. All remaining variant calls were merged into a single meta-alignment file. The meta-alignment of informative core SNP positions was used to create a maximum likelihood phylogenetic tree using PhyML (version 3.0) with generalized time reversible model [35] using parameters: Evolution model = “GTR”, Branch support = “SH-like aLRT” and Tree topology search operation = “Best of NNI and SPR.” A gamma model was used with the number of categories set to “4” and shape parameter to “e.” The proportion of invariant sites was set to 0.0. The phylogenetic tree was visualized using FigTree [http://tree.bio.ed.ac.uk/software/figtree/] and rooted on *S*. *pneumoniae* R6 based on its historical importance of being broadly used in molecular studies, as well as its sufficient evolutionary distance from the non-capsule switch serotype 22F isolates. The tree topology was validated using IQTree [36] which determined TVM the best evolutionary model, and with ASC correction was applied an essentially identical tree was produced (S2 Fig). Phylogenetic clades were determined by cluster analysis using ClusterPicker [37] with the following settings: initial and main support thresholds = 0.9, genetic distance threshold = 0.040 and the large cluster threshold = 10.

### Molecular typing

WGS data were used to identify the presence of macrolide (*ermA*, *ermB*, *ermC*, *ermF*, *mefA/E)*; tetracycline (*tetM*); trimethoprim-sulfamethoxazole (*folA*, *folP*) and penicillin (*pbp1a*, *pbp2b*, *pbp2x*) molecular antimicrobial resistance (AMR) markers [8,28,38–40] and to detect virulence factors *pspA* (pneumococcal surface protein A), *pspC* (pneumococcal surface protein C), *ply* (pneumolysin), *pavA* (pneumococcal adhesion and virulence A), *lytA* (autolysin A), *phtA*, *phtB*, *phtD*, *phtE* (polyhistidine triad complex A, B, D, E, respectively), *nanA*, *nanB*, *nanC* (neuraminidase A,B,C, respectively), *rrgA* (pilus-1), *sipA* (pilus-2), *pcpA* (pneumococcal choline binding protein A) and *psrp* (pneumococcal serine-rich protein) [41,42]. The presence or absence of molecular marker genes in the isolates were determined by querying reference nucleotide sequences against assembled contig files using BLAST [43] with the e-value cutoff option set to 10e-100. Penicillin binding protein (PBP) amino acid allelic profiles were determined as described by Metcalf et al [28]. The number of 23S rRNA allele mutations were determined by the SNVPhyl workflow using an allele of *S*. *pneumoniae* R6 (locus tag sprr02) as a mapping reference and interrogating the allele counts at nucleotide positions 2061 from the resultant variant call files (.vcf). MLST allelic profiles determined in silico and queried using the PubMLST *S*. *pneumoniae* MLST website (http://pubmlst.org/spneumoniae/) sited at the University of Oxford to determine a sequence type (ST).

The accessory genome was analyzed using Neptune DNA signature discovery software [44] and pangenome analysis using GView [45].

### Statistical analysis

The measures of association between values for characteristic differences were determined by χ^2^ or Fisher exact test with two-tailed p values of <0.05 at 95% confidence considered significant. Odds ratios and confidence intervals were calculated using 95% confidence limits.

## Results

### Isolates

Of the 112 Canadian invasive serotype 22F isolates, 48 (43%) were from females, 61 (55%) were from males, and 3 (2%) had no gender provided. The patient ages were available for 111 isolates and ranged from 20 weeks to 94 years with median and average age of 51 and 58 years, respectively. Children <5 years of age accounted for 18% (*n* = 20), those 5–15 years for 6% (*n* = 7), 16–64 years for 33% (*n* = 37), and ≥65 years for 42% (*n* = 47) of the isolates. Invasive isolates from sterile clinical isolation sites included blood (*n* = 96), cerebrospinal fluid (*n* = 9), pleural fluid (*n* = 4), synovial fluid (*n* = 1), spleen (*n* = 1) and adenoid tissue (*n* = 1). Non-invasive isolates were from respiratory sources (*n* = 25). Enhanced patient information such as vaccine history, disease severity, co-morbidities and outcomes were not available.

### Whole genome sequencing

Illumina MiSeq sequencing yielded an average 716,179 reads/genome and average genome coverage was 102X. De novo assembly resulted in an average of 37 contigs per isolate and an average contig and N50 length of 57,109 and 97,718 nucleotides, respectively. The percentage of valid and included positions in the core genome was 88.7% and 29,057 sites were used to generate the core SNV phylogeny.

### Phylogenomic analysis

**Fig 1:**
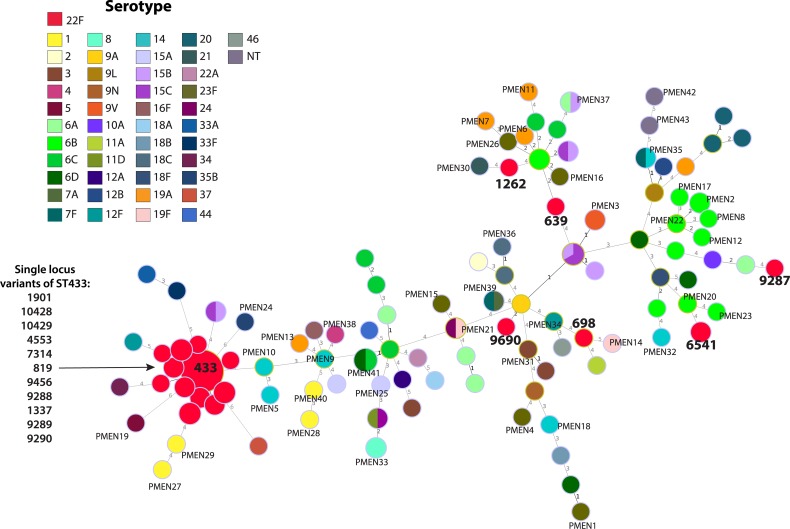
Multi-locus sequence type (MLST) comparison of the genetic relatedness of *Streptococcus pneumoniae* serotype 22F. Node colour indicates serotype and diameters are proportional to the number of isolates. Major MLST types of serotype 22F isolates are displayed in bold text and Pneumococcal Molecular Epidemiology Network (PMEN) clones are indicated. Branch labels are the number of allelic variations between sequence types; branch lengths are not to scale.

**Fig 2:**
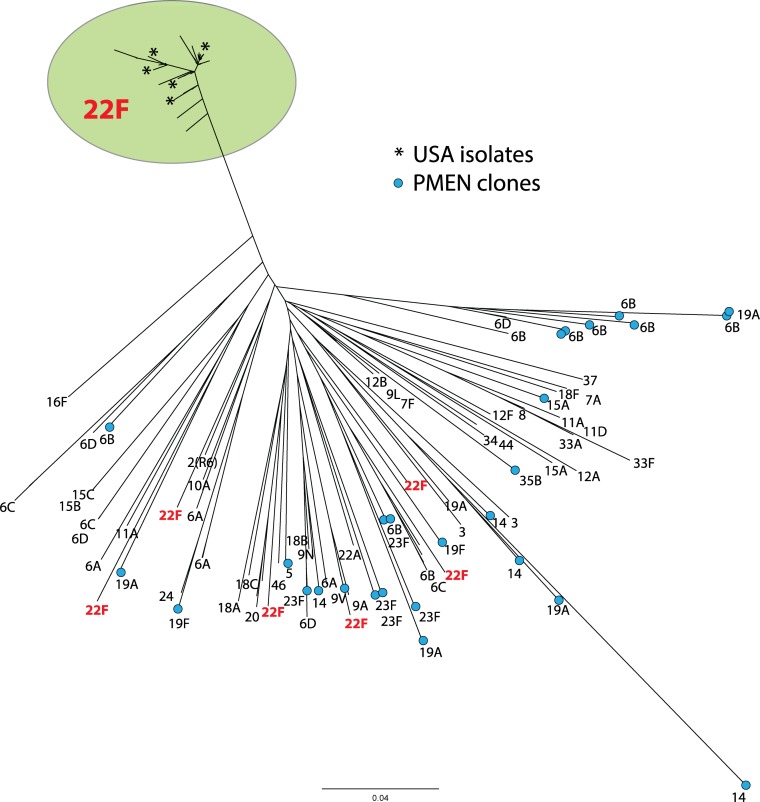
Core SNV phylogenetic comparison of genetic relatedness of *Streptococcus pneumoniae* serotype 22F. Node labels indicate serotype, an asterisk indicates USA strains and blue nodes indicate Pneumococcal Molecular Epidemiology Network (PMEN) clones. The length of the scale bar represents the estimated evolutionary divergence between isolates on the basis of average genetic distance between strains (estimated number of substitutions in the sample / total number of high quality SNVs).

Similar genetic relationships were observed using both MLST and whole-genome phylogenetic analyses on Canadian, USA and PMEN strains. *S*. *pneumoniae* serotype 22F genomes formed a large, tightly clustered, serotype 22F/ST433 clonal complex (CC), with the exception of six outlier Canadian 22F isolates that were located distantly among the other miscellaneous serotypes and PMEN genomes (Figs 1 and 2). The 22F/ST433 CC included ST433 (*n* = 113), and single locus variants (SLVs) ST819 (*n* = 1), ST4553 (*n* = 4), ST7314 (*n* = 2), ST9288 (*n* = 1), ST9289 (*n* = 4), ST9290 (*n* = 1), ST9456 (*n* = 2), ST10428 (*n* = 1) and ST10429 (*n* = 2); while the six outlier 22F isolates consisted of singleton ST369, ST698, ST1262, ST6541, ST9287 and ST9690 (Fig 1).

**Fig 3:**
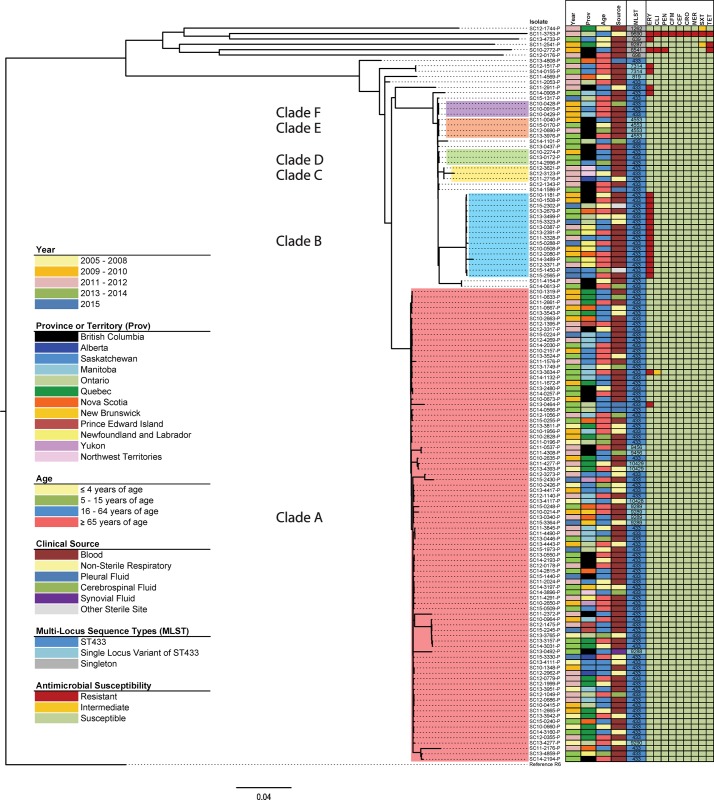
Whole genome core SNV maximum likelihood phylogenetic tree of 137 *Streptococcus pneumoniae* serotype 22F isolates collected in Canada from 2005–2015. The maximum likelihood phylogenetic tree is rooted on the reference genome of *S*. *pneumoniae* R6 (GenBank accession no. NC_003098.1) and the scale bar represents the estimated evolutionary divergence between isolates on the basis of average genetic distance between strains (estimated number of substitutions in the sample / total number of high quality SNVs). Clades A through F identified by cluster analysis are denoted with shading. Coloured columns in the right side heatmap represent: year of isolation (Year); province or territory isolated (Prov); patient age group (Age); clinical isolation source (Source); multi-locus sequence type (MLST); and antimicrobial susceptibilities to erythromycin (ERY), clindamycin (CLI), penicillin (PEN), cefepime (CFM), cefotaxime (CEF), ceftriaxone (CRO), meropenem (MER), trimethoprim/sulfamethoxazole (SXT) and tetracycline (TET).

Core SNV phylogenetic cluster analysis of the 137 Canadian invasive and non-invasive *S*. *pneumoniae* serotype 22F genomes grouped the isolates into 6 clades (*n* = 117) with 20 heterogeneous isolates outside of these 6 lineages (Fig 3). Cluster analysis using a genetic distance threshold of 4.5% produced 2 major clades; however selecting a lower threshold of 4.0% further sub-grouped one of the clades into 5 lineages (clades B–F). A group of six phylogenetically distant outlier isolates with singleton MLST types had an average of 9,154 SNVs from the 22F/ST433 CC strains (S3 Table), and there was a maximum of 9,866 SNVs between the two outlier isolates SC10-2772-P and SC11-2911-P. Clade A (*n* = 88) was the largest and most diverse lineage with an average of 203 SNVs between isolates, whereas clade B (*n* = 16) had an average of 39 SNVs (S3 Table). Four other smaller clades were identified consisting of 3 to 4 isolates each (Fig 3).

No clustering was observed by age group, gender, or clinical source. All isolates collected prior to 2009 (*n* = 11) were located in clade A. One isolate from 2009 appeared in clade F; and another was among the 6 outlier strains. All other isolates of clades B–F, as well as the remainder of the heterogeneous isolates outside the major lineages had more recent collection dates ranging from 2010–2015. Regional clustering was seen in clade B, where 6 (38%) of 16 isolates were from Newfoundland and Labrador (p<0.05, OR = 35.7, CI = 6.4–200.4), and in the smaller clades C, D, E and F that were associated with the Northwest Territories, British Columbia or Manitoba.

### Antimicrobial resistance

ERY-R was observed in 2 isolates of clade A, all 16 strains of clade B, and in 7 other isolates located outside the major phylogenetic lineages. Two outlier ERY-R/CLI-R isolates had *ermB*, and one ERY-R/CLI-I isolate in clade A had an A2061G mutation in all four 23S rRNA alleles. The remaining 22 ERY-R isolates possessed *mef* within the 5.5 kb chromosomal insertion element denoted as the macrolide efflux genetic assembly (mega) element [40]. No isolate simultaneously possessed both *ermB* and *mef*. Also present among the phylogenetic outliers were 3 TET-R isolates (MIC >8 μg/ml) containing *tetM*; 1 SXT-R (MIC >4/76 μg/ml) isolate with the FolA isoleucine (I) to leucine (L) substitution at amino acid position 100 (I100L) and a FolP glycine-serine insertion at positions 60 and 61, respectively; 1 SXT-I (MIC = 1/19 μg/ml) isolate with glycine-arginine FolP 60/61 insertion only; and another SXT-I (MIC = 2/38 μg/ml) isolate with the FolA I100L determinant and an arginine-proline FolP 60/61 insertion. Also present among the outlier strains were a PEN-R (MIC = 0.12 μg/ml) isolate with recombinant *pbp2b* and *pbp2x* and the only MDR isolate (ERY-R, CLI-R, PEN-R, CFM-R, CEF-R, CRO-R, MER-R, SXT-R and TET-R) with a 15:12:18 PBP allelic profile (S3 Fig) [28].

### Virulence factors

All Canadian serotype 22F isolates contained *lytA*, *pspC*, *ply*, *pavA*, *pcpA*, *phtA*, *phtB*, *phtD*, *phtE*, *pspA*, and *nanA*; and none had *sipA* (pilus-2). Factor *nanB* was present all isolates analyzed except one phylogenetic outlier (Fig 3). The MDR phylogenetic outlier was the only isolate that contained *rrgA* (pilus-1); and *nanC* was present only in another outlier. More heterogeneity was observed with *psrp* found in 3 isolates all also located outside the major lineages.

Accessory genome analysis (S4 Fig) identified several sequences of a clonal nature including hypothetical proteins (GenBank IDs AKU19949.1 and AKU19950.1), an acetyltransferase (ADM85197.1) and endo-beta-N-acetylglucosaminidase (CAR68298.1) present in the majority of 22F strains but absent from all isolates of clade B. Conversely, a cluster of PTS system genes (AFC95208.1, ACA37579.1, ACA35669.1, ACA36344.1, ACA36574.1, AFC95205.1, ACA37114.1) and a hypothetical protein (CBW37054.1) were present primarily only in clade B isolates and absent from the other strains. A further group of accessory genes were absent from clade A and present in strains of clades B–F included phage-related proteins (LK020690.1, LK020690.1, CCM08136.1, CDQ30157.1, AAK75017.1); branched-chain amino acid permeases (ACO22328.1, ACO23989.1); hypothetical proteins (ADM83788.1, AOG57188.1); a lantibiotic biosynthesis protein (AJD71099.1); an IS515 transposase-like protein (ADM83793.1); asparagine synthase family proteins (AOG57189.1, AOG57190.1) and a Tn*916*-type conjugative element containing the mega element (FR671415.1).

## Discussion

*S*. *pneumoniae* serotype 22F has increased in prevalence in Canada. Both MLST and WGS analysis showed that Canadian and USA isolates examined were genetically very homogenous forming a large closely related cluster, with 6 outlier isolates located phylogenetically distantly among the other serotypes and PMEN clones (Figs 1 and 2). This tight clustering suggests an evolutionarily young group of organisms that has recently emerged and not yet had time to substantially diversify. The 22F/ST433 CC is the predominant MLST clone in Canada and other countries including the UK, Portugal, Spain, Japan and the USA [7,20,25,28,46–48]. Another major clone reported in the UK is the 22F/ST698 CC [20] that is otherwise relatively rare in this study as well as in the USA and Brazil [28,49] highlighting a difference in the populations of 22F in the Americas versus Europe. The PubMLST pneumococcal isolate database associates the phylogenetic outlier ST1262 with serotype 15B/C; ST639, ST6541 and ST9287 with serotypes 6A/B/C; and ST9690 (a double locus variant of ST156 associated PMEN clone Spain^9V^-3) with serotype 9V. The relatively heterogeneous nature of the MLST profiles, molecular profiles and phenotypic results among this group of Canadian phylogenetic outlying 22F strains (Fig 3) is supportive of capsule switching events having occurred.

Despite the high degree of similarity among Canadian *S*. *pneumoniae* serotype 22F isolates, core SNV phylogenetic analysis successfully resolved the large 22F/ST433 CC into 6 lineages with SLVs grouping into subclades. Older isolates selected for this study collected prior to 2009 appeared only in clade A, which suggest it as a potential ancestral lineage to the relatively more recent clades B to F. Regional clustering was seen in clade E consisting entirely of isolates from British Columbia, clade C associated with isolates from Northwest Territories, and clade F with Manitoba, suggesting potential local transmission events associated with invasive disease. A clonal expansion of ERY-R/*mef* strains (clade B) was observed to originate in the eastern provinces of Newfoundland/Labrador and Nova Scotia in 2010, and subsequently identified in Ontario, Saskatchewan, and British Columbia (Fig 3).

Although the conjugate vaccines have been successful in reducing disease prevalence caused by antimicrobial-resistant strains [4], the emergence and spread of antimicrobial-resistant non-vaccine serotypes is a continual threat. The major molecular AMR determinants in *S*. *pneumoniae* have been identified, many of which are acquired through the exchange of genetic content with commensal organisms in the human nasopharynx to create successful antimicrobial-resistant clonal lineages [8,27,28]. The overall low level of AMR among *S*. *pneumoniae* serotype 22F in this study reflects current observations worldwide [6,12,21,28,46–48].

Macrolide-resistance in North America and the UK has been predominantly associated with the *mef* efflux transport system conferring low level ERY-R, whereas in Europe modifications to ribosomal methylase *ermB* is prevalent giving high-level resistance to both ERY and CLI [8,40]. In this study, ERY-R/CLI-S was associated with *mef* and was mainly confined to a recent expanding clonal lineage (clade B), while two ERY-R/CLI-R phylogenetic outlier isolates had *ermB*. One ERY-R/CLI-I isolate had the A2061G (A2059G in *E*. *coli*) point mutation in all four 23S rRNA alleles, which also has been shown to decrease macrolide susceptibility [8,50]. Decreased susceptibilities to other antimicrobials were restricted to four phylogenetic outlier isolates. Interestingly the 22F/ST9690 MDR isolate had a PBP allelic profile (15:12:18) identical to that of a similarly MDR 9V/ST156 strain (a double locus variant of ST9690) reported in the USA by Metcalf et al [28], suggesting that the former isolate acquired a 22F CPS locus through a capsule switch event. Further correlations of molecular markers to phenotypic resistance were observed with an SXT-I isolate with a FolP di-amino acid insertion, and a fully resistant isolate that had both the FolP insertion as well as the FolA I100L substitution [8,28]. Another isolate with both resistance determinants was not fully resistant (MIC = 2/39 μg/ml).

A wide range of virulence factors are involved in pneumococcal pathogenesis, however their direct roles in disease have not been fully resolved. Virulence among pneumococci is complicated by variable capacities of strains to produce virulence factors, the presence of a large proportion of as yet functionally unassigned hypothetical proteins, and the multifunctional and complementary mechanisms involved in the expression of virulence determinants. Analyses of the distribution of virulence factors may provide valuable insight into the evolution and dissemination of strains causing severe disease. The prevalence of virulence factors among the Canadian serotype 22F strains are similar to previous reports where *nanA*, *ply*, *pavA*, *lytA*, *pspC*, *phtA*, *phtE*, *pcpA* and *pspA* have been reported as being almost ubiquitously present; whereas *nanB*, *nanC*, *psrp*, and *rrgA* (pilus-1) were more variable [41]. Virulence factors and invasiveness have been described to have a close association with genotype [41]; consequently, the high degree of homogeneity among the relatively recently emerged Canadian serotype 22F strains is not unexpected. The accessory genome contained proteins that were present primarily in the younger lineages (clades B–F) and included an assortment of phage proteins, permeases, and biosynthetic proteins for which functional significance remains unclear.

Limitations of this study include the relatively small number of isolates collected prior to 2010 before the implementation of widespread national surveillance of IPD, limiting further exploration of the ancestral and evolutionary background of serotype 22F strains in Canada. The limited availability of international serotype 22F genomes restricted the comparisons to only Canadian and USA strains, which owing to close geographical proximity resulted in limited diversity. Other caveats of this study include the lack of data for disease severity and patient outcome, which could provide better measures of bacterial invasiveness.

*S*. *pneumoniae* serotype 22F in Canada are genetically highly similar, however whole-genome sequence analysis provided increased resolution of the large 22F/ST433 CC. Temporal and regional clustering identified the emergence of a successful ERY-R clone in Eastern Canada post-2010 and identified potential regional transmission events in Western Canada. Although the observed capsule switch events have occurred, such as to an MDR clone, no such lineages are currently expanding in Canada.

Despite the success conjugate vaccines have achieved in lowering invasive pneumococcal disease attributed to constituent serotypes in many countries, successfully expanding lineages of pneumococci that evade vaccines by serotype replacement from vaccine pressure, capsule switching or the acquisition of virulence factors represent a direct threat to public health. The ability to rapidly recognize and identify these clinically important emerging serotypes is critical to inform public health, prevention and control strategies. Whole-genome sequencing technology used in a genomic epidemiology approach is redefining pathogen surveillance, transmission analysis, outbreak response and diagnosis to provide tools that rapidly recognize these emergent threats to public health, prevention and control.

## Supporting information

S1 Fig Serotype trends of invasive *Streptococcus pneumoniae* isolated in Canada, 2010–2014. (TIF)

S2 Fig Validation of whole genome core SNV maximum likelihood phylogenetic tree of 137 *Streptococcus pneumoniae* serotype 22F isolates collected in Canada from 2005–2015. The maximum likelihood phylogenetic tree was generated with IQTree [36] using the TVM+ASC evolutionary model and is rooted on the reference genome of *S*. *pneumoniae* R6 (GenBank accession no. NC_003098.1). The scale bar represents the estimated evolutionary divergence between isolates on the basis of average genetic distance between strains (estimated number of substitutions in the sample / total number of high quality SNVs). Clades A through F were identified by cluster analysis of the phylogenetic tree depicted in Figs. 3 and S4 created using the GTR evolutionary model without ASC correction.
(EPS)

S3 Fig Alignment of penicillin binding protein (Pbp) 1a, 2b and 2x alleles. Sequence position 1 corresponds to amino acid positions 371, 379 and 229 of Pbp1a, Pbp2a and Pbp2x respectively. Allele 0 for each protein from Metcalf et al [28] and asterisk beside the allele ID indicates a novel allele.
(EPS)

S4 Fig Accessory genome of *Streptococcus pneumoniae* serotype 22F isolated in Canada. Whole genome core SNV maximum likelihood phylogenetic tree of 137 *Streptococcus pneumoniae* serotype 22F isolates collected in Canada from 2005–2015, rooted on the reference genome of *S*. *pneumoniae* R6 (GenBank accession no. NC_003098.1). The length of the scale bar represents the estimated evolutionary divergence between isolates on the basis of average genetic distance between strains (estimated number of substitutions in the sample / total number of high quality SNVs). Clades A through F identified by cluster analysis are denoted with shading. Accessory DNA sequence signatures determined using Neptune DNA signature discovery software [44] and pangenomic analysis using GView [45]. Blue and grey squares indicate presence and absence of signature DNA sequence, respectively. Green highlighted signature IDs correspond with phage associated proteins.
(EPS)

S1 Table Geographical and temporal distribution of Canadian invasive *Streptococcus pneumoniae* serotype 22F isolates selected for phylogenetic analysis. (DOCX)

S2 Table Geographical and temporal distribution of Canadian non-invasive respiratory *Streptococcus pneumoniae* serotype 22F isolates selected for phylogenetic analysis. (DOCX)

S3 Table Number of single nucleotide variations (SNVs) in the core genome within, and between, the major phylogenomic clades of Canada *Streptococcus pneumoniae* serotype 22F isolates. (DOCX)

S1 File Isolate metadata for phylogenetic analysis of emergent *Streptococcus pneumoniae* serotype 22F causing invasive pneumococcal disease using whole genome sequencing. (XLSX)
