# Supplementary figures and images for: Phylogenetic analysis of emergent Streptococcus pneumoniae serotype 22F causing invasive pneumococcal disease using whole genome sequencing
Source: PLoS One. 2017 May 22;12(5):e0178040. doi: 10.1371/journal.pone.0178040 (PMC5439729; doi:10.1371/journal.pone.0178040)

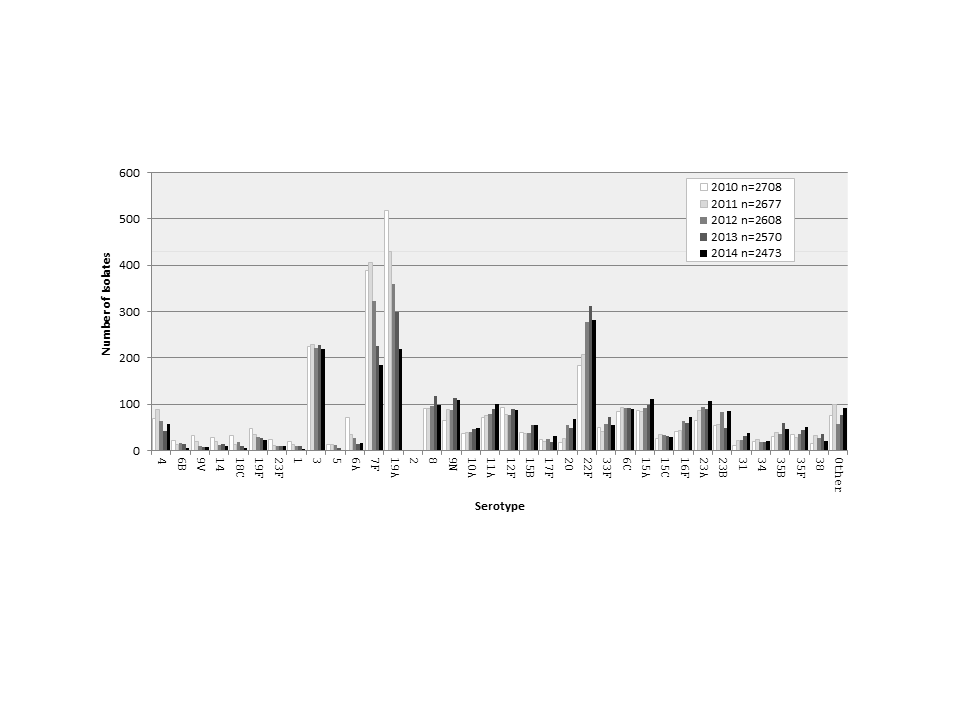

Supplement: S1 Fig — (TIF) [file pone.0178040.s001.tif]
