# Supplementary material for: Phylogenetic analysis of emergent Streptococcus pneumoniae serotype 22F causing invasive pneumococcal disease using whole genome sequencing
Source: PLoS One. 2017 May 22;12(5):e0178040. doi: 10.1371/journal.pone.0178040 (PMC5439729; doi:10.1371/journal.pone.0178040)
Supplement: S1 Table — (DOCX) [file pone.0178040.s005.docx]

**S1 Table. Geographical and temporal distribution of Canadian invasive *Streptococcus pneumoniae* serotype 22F isolates selected for phylogenetic analysis**

|  | Year Isolated | | | | | | |  |
| --- | --- | --- | --- | --- | --- | --- | --- | --- |
| Province/Territory | 2005 – 2009^a^ | 2010 | 2011 | 2012 | 2013 | 2014 | 2015 | Total |
| British Columbia | 0 | 6/16^b^ | 4/34 | 5/40 | 6/44 | 6/49 | 1/32 | 28/215 |
| Saskatchewan | 1 | 2/8 | 3/8 | 1/10 | 2/6 | 2/9 | 2/9 | 13/50 |
| Manitoba | 1 | 3/7 | 2/3 | 4/14 | 3/11 | 3/9 | 1/6 | 17/50 |
| Ontario | 1 | 1/81 | 1/94 | 1/124 | 1/124 | 2/108 | 3/105 | 10/636 |
| Quebec | 3 | 5/27 | 3/28 | 3/30 | 1/62 | 1/51 | 0/42 | 16/240 |
| New Brunswick | 1 | 0/8 | 0/7 | 0/6 | 0/9 | 0/9 | 0/11 | 1/50 |
| Prince Edward Island | 0 | 0/0 | 0/0 | 2/2 | 0/1 | 0/1 | 1/4 | 3/7 |
| Nova Scotia | 0 | 2/3 | 2/3 | 1/4 | 2/4 | 4/12 | 0/9 | 11/35 |
| Newfoundland | 0 | 1/3 | 1/3 | 2/5 | 2/3 | 1/2 | 1/2 | 8/18 |
| Northwest Territories | 0 | 0/0 | 0/0 | 2/2 | 0/0 | 1/1 | 0/0 | 3/3 |
| Yukon | 0 | 1/1 | 0/0 | 0/0 | 0/0 | 0/0 | 1/1 | 2/2 |
| Total | 7 | 21/183 | 16/208 | 21/278 | 17/312 | 20/282 | 10/249 | 112/1512 |

^a^Nationally representative annual denominator totals of *S. pneumoniae* serotype 22F for 2005 to 2009 are not available prior to initiation of national surveillance at the National Microbiology Laboratory, Winnipeg in 2010.

^b^Number of serotype 22F isolates selected / total serotype 22F isolates identified.
