# Supplementary material for: Phylogenetic analysis of emergent Streptococcus pneumoniae serotype 22F causing invasive pneumococcal disease using whole genome sequencing
Source: PLoS One. 2017 May 22;12(5):e0178040. doi: 10.1371/journal.pone.0178040 (PMC5439729; doi:10.1371/journal.pone.0178040)
Supplement: S2 Table — (DOCX) [file pone.0178040.s006.docx]

**S2 Table. Geographical and temporal distribution of Canadian non-invasive respiratory *Streptococcus pneumoniae* serotype 22F isolates selected for phylogenetic analysis**

|  | Year Isolated | | | | | | |  |
| --- | --- | --- | --- | --- | --- | --- | --- | --- |
| Province | 2007 – 2009^a^ | 2010 | 2011 | 2012 | 2013 | 2014 | 2015 | Total |
| Alberta | 0 | 0 | 1 | 1 | 0 | 0 | 1 | 3 |
| British Columbia | 0 | 0 | 1 | 0 | 0 | 0 | 0 | 1 |
| Saskatchewan | 3 | 0 | 0 | 1 | 1 | 0 | 0 | 5 |
| Manitoba | 1 | 1 | 0 | 0 | 0 | 0 | 0 | 2 |
| Ontario | 3 | 0 | 1 | 0 | 1 | 0 | 1 | 6 |
| Quebec | 3 | 0 | 0 | 0 | 1 | 1 | 0 | 5 |
| New Brunswick | 0 | 0 | 0 | 0 | 0 | 1 | 1 | 2 |
| Nova Scotia | 0 | 1 | 0 | 0 | 0 | 0 | 0 | 1 |
| Total | 10 | 2 | 3 | 2 | 3 | 2 | 3 | 25 |

^a^Annual denominator totals for non-invasive *S. pneumoniae* serotype 22F are not available
