# Supplementary material for: Phylogenetic analysis of emergent Streptococcus pneumoniae serotype 22F causing invasive pneumococcal disease using whole genome sequencing
Source: PLoS One. 2017 May 22;12(5):e0178040. doi: 10.1371/journal.pone.0178040 (PMC5439729; doi:10.1371/journal.pone.0178040)
Supplement: S3 Table — (DOCX) [file pone.0178040.s007.docx]

**S3 Table. Number of single nucleotide variations (SNVs) in the core genome within, and between, the major phylogenomic clades of Canada *Streptococcus pneumoniae* serotype 22F isolates**

| **Clade** | **No. Isolates** | **Average SNVs within Clade** | **Maximum SNVs within Clade** | **Average SNVs**  **from next clade^a^** | **Minimum SNVs from next clade^a^** | **Maximum SNVs from next clade^a^** |
| --- | --- | --- | --- | --- | --- | --- |
| A | 88 | 203 | 1024 | 2043 | 1956 | 2612 |
| B | 16 | 39 | 191 | 800 | 713 | 1064 |
| C | 3 | 101 | 224 | 221 | 146 | 368 |
| D | 3 | 7 | 15 | 51 | 47 | 57 |
| E | 4 | 5 | 11 | 50 | 46 | 54 |
| F | 3 | 1 | 3 | 1475^b^ | 1395 ^b^ | 1911^b^ |
| Outliers^c^ | 6 | 7806 | 9785 | 9154^d^ | 8501^d^ | 9866 ^d^ |

^a^ Number of SNVs compared to the following closest ancestral clade in the phylogeny.

^b^ Number of SNVs compared to clade A in the phylogeny.

^c^ A group of six outlier isolates (SC12-1744-P, SC11-3753-P, SC13-4733-P, SC11-2541-P, SC10-2772-P, SC12-0176-P) with singleton MLST sequence types distinct from the serotype 22F/ST433 clonal complex.

^d^ Number of SNVs compared to the serotype 22F/ST433 clonal complex strains in the phylogeny (*n* = 131).
